# Non-thyroid Malignancies With Metastases to the Thyroid Gland: A Case Series and Review of the Literature

**DOI:** 10.7759/cureus.98454

**Published:** 2025-12-04

**Authors:** Anderson Okafor, Juliana Chaves de Oliveira, Moumita S Choudhury, Julie Samantray

**Affiliations:** 1 Endocrinology and Diabetes, Wayne State University, Detroit Medical Center, Detroit, USA; 2 Internal Medicine, Wayne State University, Detroit Medical Center, Detroit, USA; 3 Pathology, Wayne State University, Detroit Medical Center, Detroit, USA; 4 Endocrine Oncology, Karmanos Cancer Center, Detroit, USA; 5 Endocrinology, Diabetes, and Metabolism, Wayne State University, Detroit, USA

**Keywords:** colorectal neoplasms, melanoma, neoplasm metastasis, renal cell carcinoma, thyroid neoplasms, thyroid nodule, tumor-to-tumor metastasis

## Abstract

Metastasis of non-thyroid malignancies (NTMs) to the thyroid gland is an uncommon phenomenon. The most frequently reported primary tumors include renal cell carcinoma (RCC), lung cancer, colorectal cancer, breast carcinoma, and melanoma.

We present a case series of four patients with tumor-to-tumor metastases involving the thyroid gland. Primary tumors included colon adenocarcinoma, clear cell RCC, and malignant melanoma. Each case was evaluated for imaging findings and clinicopathologic features, treatment, and outcomes.

In all cases, the metastases occurred at a subsequent time following the primary cancer diagnosis. The average time to thyroid metastasis was 41.5 months for colorectal cancer, 68 months for RCC, and 20.9 months for melanoma. Imaging characteristics such as hypoechogenicity, irregular margins, increased vascularity, and fluorodeoxyglucose avidity were present and consistent with malignant features in all cases. Subsequent pathology analysis was essential for accurate diagnosis. Treatment consisted of hemi or total thyroidectomy in all cases, followed by thyroid hormone replacement therapy in three out of the four patients.

Although rare, metastases to the thyroid gland should be considered in patients with a history of malignancy who present with suspicious thyroid nodules. This case series demonstrates the diagnostic challenges and emphasizes the importance of radiologic vigilance and confirmatory histopathology in detecting and managing thyroid metastases from non-thyroid primaries.

## Introduction

Non-thyroid malignancies (NTMs) with metastasis to the thyroid gland are uncommon in clinical practice. Reported incidences range from 0.5% to as high as 48%. Metastases to the thyroid gland from NTMs are rare. NTMs that metastasize to the thyroid gland are renal cell, colorectal, lung, endometrium, breast carcinoma, melanoma, and sarcoma. Renal cell carcinoma (RCC) is the most common NTM that metastasizes to the thyroid gland [1,2]. Metastases of NTMs to the thyroid gland are more common in females than males (female-to-male ratio = 1.4 to 1) and in nodular thyroid glands (44.2%) [1].

Despite the thyroid gland’s rich vascularity, clinically evident metastases are rare, a phenomenon often referred to as the “thyroid vascularity paradox.” Several hypotheses have been proposed to explain this resistance, including high oxygen and iodine content, and rapid arterial blood flow that may hinder tumor cell adhesion [3].

Pre-existing thyroid conditions, such as nodular disease or thyroiditis, may create a permissive microenvironment for metastatic implantation [4]. Tumor-to-tumor metastasis, where one neoplasm serves as the host for metastatic deposits from a separate primary tumor, is exceedingly rare. The thyroid gland is an uncommon site for such events, as demonstrated in one of our cases.

We report four cases of tumor-to-tumor metastases where the recipient was a thyroid nodule and donor tumors were renal cell, colon cancer, and melanoma. We describe the clinicopathological and imaging characteristics of the metastatic thyroid lesions.

## Case presentation

Case 1: Colon cancer

A 62-year-old female presented to the ER with cramping lower abdominal pain. Imaging was concerning for thickening of the mid-descending colon. She underwent a left hemicolectomy, and histopathology revealed moderately differentiated adenocarcinoma (4.5 cm mass, full thickness, with extension to the pericolic fat, and 2/32 positive lymph nodes). Postoperative staging scans showed three sub-centimeter lung nodules. She started adjuvant therapy with FOLFOX (folinic acid, fluorouracil, and oxaliplatin).

She was known to have a heterogeneous 3.7 x 2.8 x 3.4 cm complex hyperechoic nodule on the right thyroid and a 7-mm left nodule. A fine-needle aspiration (FNA) of the right nodule prior to colon cancer diagnosis was consistent with benign cytology.

**Figure 1:**
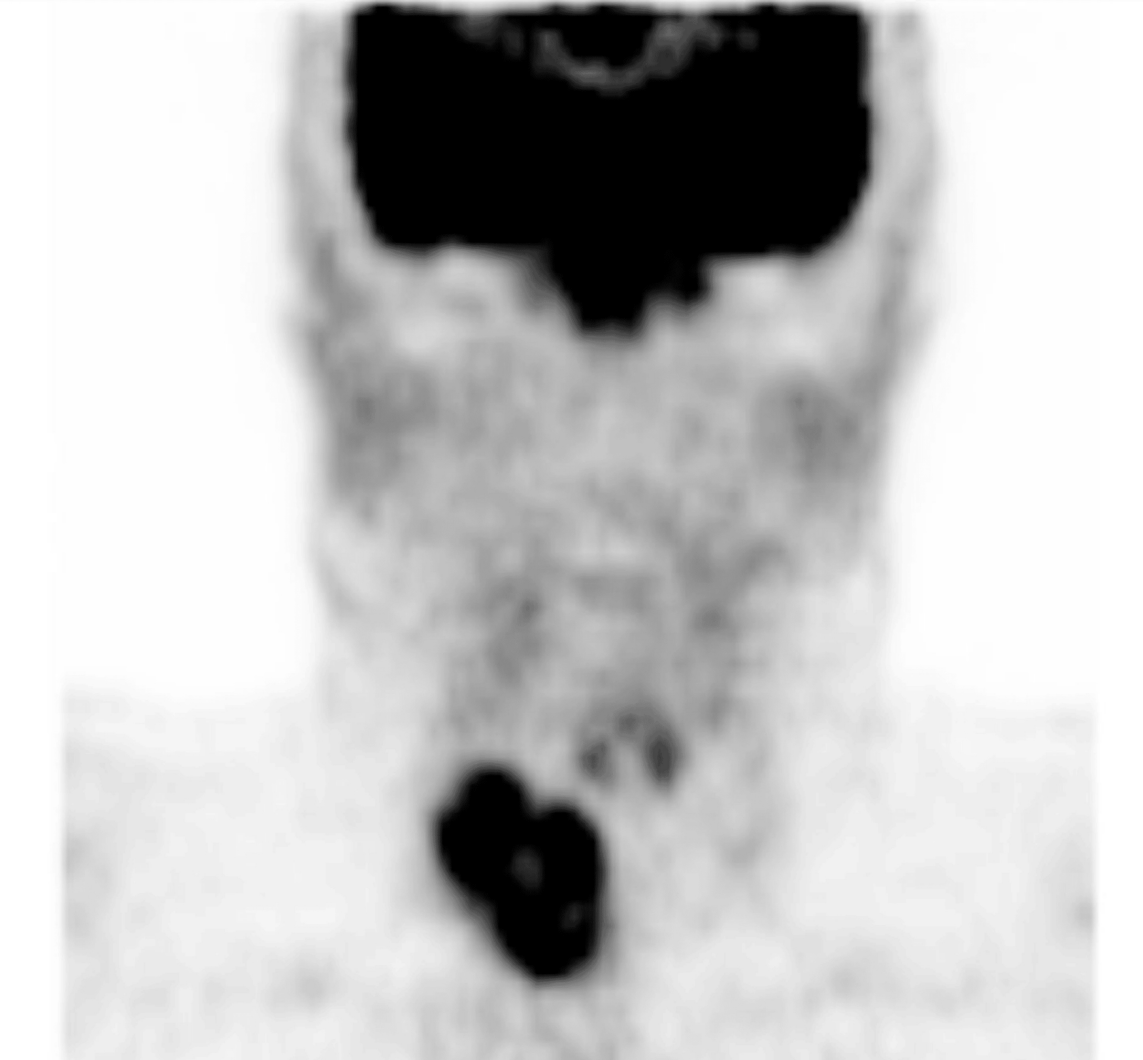
Fluorodeoxyglucose (FDG)-PET/CT demonstrating a markedly FDG-avid mass in the right thyroid lobe, measuring approximately 4.2 cm with a maximum standardized uptake value (SUVmax) of 9.3.

**Figure 2:**
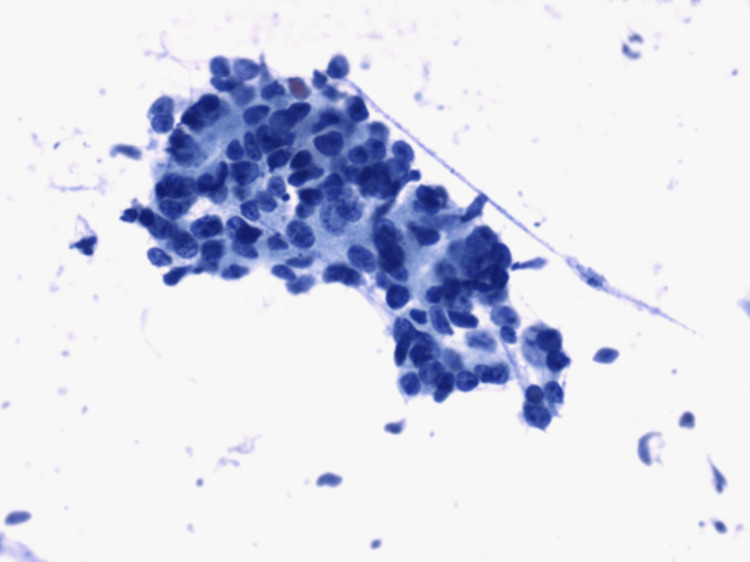
Metastatic colon adenocarcinoma metastasizing to the thyroid. Papanicolaou (PAP)-stain fine-needle aspiration (FNA) from the thyroid nodule showing pleomorphic cohesive cells consistent with metastatic adenocarcinoma.

She continued to have persistent growth in lung nodules, but she was lost to follow-up for thyroid nodules. Four years later, the right thyroid nodule had increased by more than 20% in all dimensions, and a repeat FNA was suspicious for a follicular neoplasm - Hurthle cell type. She chose to defer surgery and returned two years later. Repeat imaging revealed further size increase to 5.5 x 3.4 x 4.6 cm and was found to be fluorodeoxyglucose (FDG)-avid on PET scan (Figure 1). Two left-sided thyroid nodules were stable, with one measuring 0.7 x 0.5 x 0.6 cm (solid, hypoechoic, with good margins) and the other being a cystic nodule measuring 1.2 x 0.9 x 0.9 cm. Both left-sided nodules were non-FDG avid. She underwent a right lung, lower lobe wedge resection and right hemithyroidectomy. Thyroid pathology showed Hurthle cell adenoma with metastatic colonic adenocarcinoma (Figure 2). Thyroid function remained in the euthyroid range before and after the hemithyroidectomy.

The lung pathology was also positive for metastatic colonic carcinoma. Subsequent ultrasound of the thyroid showed a new ill-defined hypoechoic lesion in the lateral mid-left thyroid lobe, measuring 1.3 x 1.4 x 1.6 cm with internal vascularity. FNA showed metastatic adenocarcinoma, and the patient underwent a complete thyroidectomy.

Case 2: Clear cell renal cell carcinoma

**Figure 3:**
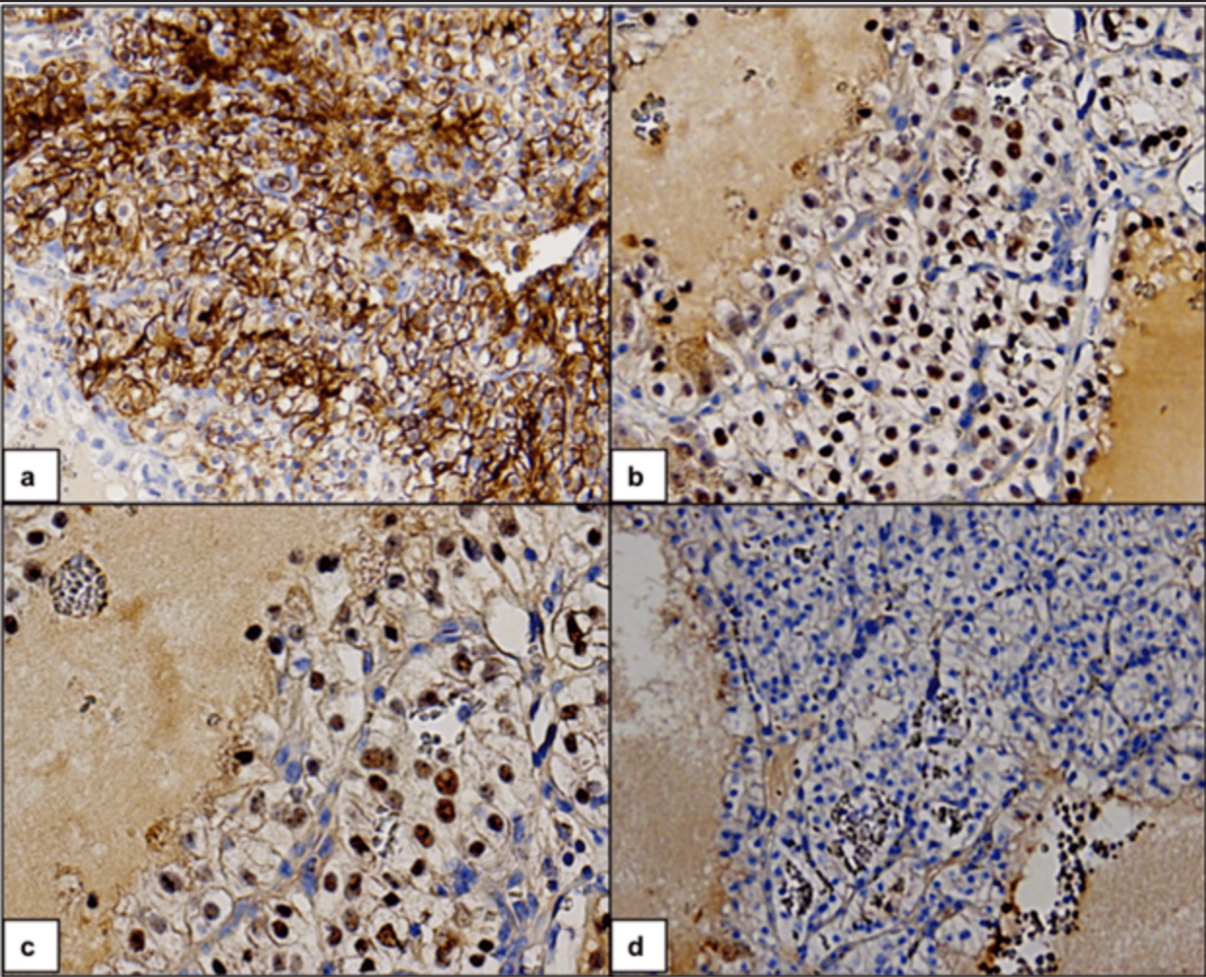
Metastatic renal cell carcinoma in the thyroid gland. Immunohistochemistry (IHC) on cell block shows cancer cells positive for (a) CA-IX (membranous), (b) PAX-8 (nuclear), and (c) PAX-2 (nuclear). (d) Cancer cells stain negative for TTF-1 (nuclear).

A 64-year-old female was diagnosed with clear cell RCC, following a nephrectomy for biopsy-proven RCC. She was noted to have a sub-centimeter right thyroid nodule on a staging scan. Two years later, a CT scan of the neck revealed the entire right lobe to be heterogeneous and bulky with irregular margins but no distinct nodule. A core biopsy of this area revealed benign cytology. A subsequent CT of the neck revealed an increase in size of the nodule to 4.7 x 2.5 cm. Ultrasound showed a hypoechoic, hypervascular, lobulated nodule with irregular margins. The left lobe was unremarkable. A right core biopsy revealed metastatic RCC (Figure 3). She underwent a right thyroidectomy and isthmectomy. Pathology showed a 4 cm mass composed of large solid nests of tumor cells with abundant clear cytoplasm and low-grade nuclei, separated by delicate vasculature, consistent with metastatic clear cell RCC. She developed hypothyroidism after the hemithyroidectomy and was started on levothyroxine. She continues to receive maintenance therapy with pazopanib, with no evidence of metastatic disease.

Case 3: Clear cell renal cell carcinoma

A 53-year-old female was diagnosed with right RCC and underwent right nephrectomy. On a staging scan, she was found to have bilateral thyroid nodules and a lesion in her pancreas. Biopsies revealed metastatic RCC in the pancreas and benign cytology of a 1.1 cm right thyroid nodule. She underwent pancreatectomy, splenectomy, and total abdominal hysterectomy/bilateral salpingo-oophorectomy (TAH/BSO). Pathology revealed clear cell carcinoma involving the right fallopian tubes, ovaries, pelvic mass, body, and tail of the pancreas, with negative margins. The patient declined adjuvant tyrosine kinase inhibitor therapy. One-year follow-up ultrasound of the thyroid showed a new 4 cm isthmus nodule. FNA of this nodule showed indeterminate cytology (Bethesda III). Molecular testing resulted as suspicious. She underwent right thyroid lobectomy and isthmectomy. Pathology revealed metastatic clear cell RCC. The patient declined further oncology treatment. She is currently on levothyroxine.

Unfortunately, the imaging for this case could not be accessed for inclusion in this manuscript.

Case 4: Melanoma

**Figure 4:**
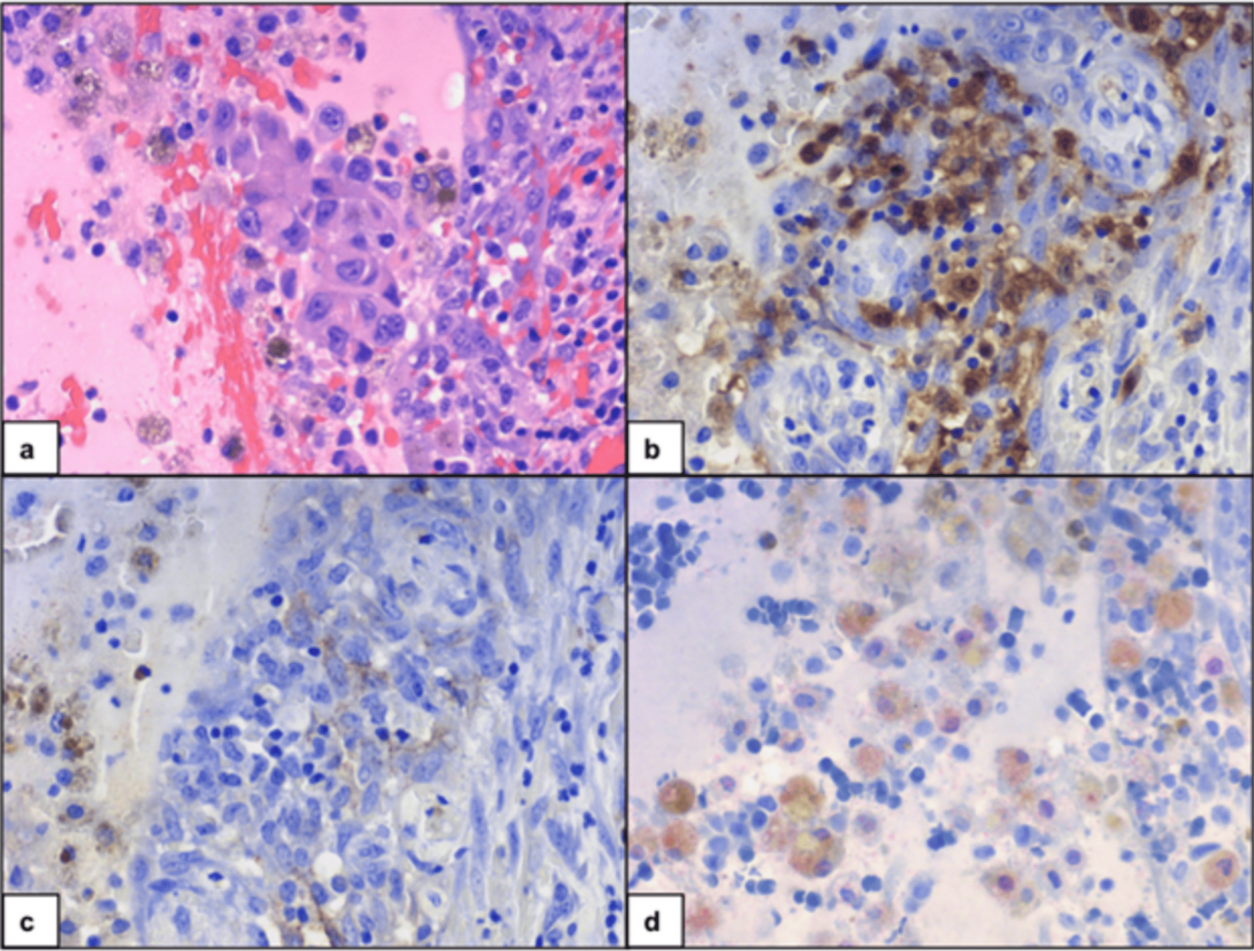
Melanoma metastasis to the thyroid. (a) Cell block showing malignant melanocytes. Melanoma cells are immunoreactive for (b) S-100 and (c) HMB-45, and (d) focally immunoreactive with MITF.

A 25-year-old male with BRAF V600E mutation-positive malignant melanoma of the right eyebrow underwent a wide local excision and sentinel lymph node biopsy. Two parotid lymph nodes were microscopically involved by tumor, but there was no evidence of distant metastases. He was treated with adjuvant nivolumab therapy for a year. Surveillance FDG PET revealed minimal FDG avidity (SUV) in the right thyroid lobe with corresponding vague hypodensity. An ultrasound of the thyroid showed a 1.2 x 0.8 x 0.9 cm hypoechoic, solid right thyroid nodule corresponding to the PET-positive findings. There was also a solid, hypoechoic 0.8 x 0.6 x 0.8 cm left inferior nodule. FNAs of both nodules were positive for malignant cells. He underwent a total thyroidectomy with right selective neck dissection, levels 2 through 4. The final pathology on the right was positive for metastatic melanoma (Figure 4), with an incidental focus of micro-papillary thyroid carcinoma (PTC). The sub-centimeter, similar appearing, hypoechoic nodule in the left thyroid lobe was also positive for micro-PTC. He is on dabrafenib and trametinib, with no evidence of metastatic disease.

## Discussion

Metastatic involvement of the thyroid gland is an uncommon clinical finding, and the phenomenon of tumor-to-tumor metastasis, when a donor tumor metastasizes into a recipient neoplasm, is exceedingly rare. In our case series, we observed this unusual event, exemplified by case 1, in which a metastatic lesion was identified within a previously cytology-proven benign thyroid nodule. Metastases to the known thyroid nodule were seen four years after the diagnosis of colon cancer, consistent with metachronous lesions described. Lung metastases were discovered around the same time as the first thyroid metastatic lesion. Keranmu et al. reported that 81.0% of patients with metastatic colorectal cancer to the thyroid showed concomitant lung metastasis [5]. Cases 2 and 3 present as metachronous metastases from RCC in previously cytologically-proven benign nodules. RCC accounts for almost 48% of NTMs that metastasize to the thyroid [1]. Both synchronous and metachronous metastases from RCC to the thyroid have been reported [6]. Metastatic melanoma to the thyroid is much less frequent and accounts for 4% of metastases to the thyroid [1,2]. Interestingly, melanoma and thyroid cancer share a common genetic alteration, such as the BRAF V600E mutation. The use of immunohistochemistry is key to the cytopathologic diagnosis of these lesions.

There are two proposed hypotheses for metastases to the thyroid. One suggests that the thyroid’s rich vascularity makes it an ideal environment for tumor metastases. Another hypothesis is that a diseased thyroid, such as in conditions like Hashimoto’s thyroiditis and nodular thyroid, may be more vulnerable to metastases due to decreased blood flow, oxygen levels, and iodine availability. None of the four cases in this study demonstrated lymphocytic thyroiditis in their final pathology. Additionally, except for the patient with metastatic colon cancer, none of the patients had previously known thyroid nodules.

To diagnose a tumor-to-tumor metastasis, the following criteria must be met [7,8]: (1) more than one primary tumor must exist; (2) the recipient tumor must be a true benign or malignant neoplasm; (3) the donor malignancy must be a true metastasis; and (4) the exclusion of contiguous growth of one tumor into another adjacent tumor (also known as a “collision tumor”), embolization of tumor cells, or metastasis to the lymphatic system, particularly if it had already been involved by generalized lymphatic or hematological malignancy.

It is important to note that the imaging features of the thyroid lesions in these cases exhibited common signs of malignancy. In case 1 (colon cancer), the thyroid nodule was initially heterogeneous and hyperechoic (present-day TIRADS (Thyroid Imaging Reporting and Data System) 3), later evolving into an FDG-avid lesion with ill-defined margins and internal vascularity. Case 2 (RCC) presented a hypoechoic, hypervascular nodule with irregular margins (present-day TIRADS 4), and case 3 also revealed solid, hypoechoic nodules (present-day TIRADS 4) that were suspicious for RCC metastasis. In case 4 (melanoma), the nodule appeared solid hypoechoic (TIRADS 4) and minimally FDG-avid. The features observed in these cases, including growth, irregular borders, hypoechogenicity, increased vascularity, and FDG avidity, strongly suggest metastatic involvement rather than benign thyroid disease. This underscores the importance of carefully evaluating thyroid nodules in patients with a history of non-thyroid malignancies. A summary of the clinicopathologic and imaging characteristics of the four cases is presented in Table 1.

**Table 1:** Clinical characteristics and outcomes of patients with thyroid metastases from primary cancers. RCC: renal cell carcinoma; a-TPO: anti-thyroid peroxidase antibody; ATG: anti-thyroglobulin.

Cases	Primary cancer	Thyroid nodule needle biopsy	Symptoms	Thyroid status	Time from primary cancer diagnosis to metastasis	Metastatic sites	Treatment of thyroid mass	Outcome after thyroid surgery
Case 1	Colon	Metastatic adenocarcinoma	None	Bilateral nodules, euthyroid, negative a-TPO	41.5 months	Bilateral thyroid, lungs	Total thyroidectomy	Succumbed to colon cancer
Case 2	Clear cell RCC	Metastatic RCC	Mild dysphagia	Solitary nodule, euthyroid, negative a-TPO	~68 months	Unilateral thyroid	Hemi-thyroidectomy	L-thyroxine. No evidence of metastatic disease
Case 3	Clear cell RCC	Metastatic clear cell RCC	None	Bilateral nodules, euthyroid, unknown a-TPO	98 months	Bilateral multifocal thyroid, pancreas, fallopian tubes, ovaries	Total thyroidectomy	L-thyroxine. Lost to follow-up
Case 4	Melanoma	Positive for malignant cells	None	Bilateral nodules, euthyroid, negative a-TPO, negative ATG	20.9 months	-	Total thyroidectomy	L-thyroxine and targeted therapy

As summarized in Table 1, the average time from NTM diagnosis to the detection of thyroid metastasis was 41.5 months for colorectal cancer, 68 months for RCC, and 20.9 months for malignant melanoma. The findings in this case series align with trends seen in the literature [5], though some variability exists in the time to thyroid metastasis [8]. The time intervals for colon cancer and RCC are consistent with reported trends, while the melanoma case showed a faster progression. This suggests variability in disease progression, which may be influenced by individual patient factors, treatment regimens, or monitoring methods. Further research with larger sample sizes could help clarify these trends.

## Conclusions

Metastases to the thyroid from NTMs are rare but clinically relevant findings. This case series demonstrates the diagnostic challenges and emphasizes the importance of radiologic vigilance and confirmatory histopathology in detecting and managing thyroid metastases from non-thyroid primaries. Recognition of the rare phenomenon of tumor-to-tumor metastasis further expands the diagnostic spectrum of thyroid lesions and reinforces the need for careful assessment. Larger studies are needed to better define the clinical course and prognostic implications of thyroid metastases from NTMs.
